# Inter-laboratory mass spectrometry dataset based on passive sampling of drinking water for non-target analysis

**DOI:** 10.1038/s41597-021-01002-w

**Published:** 2021-08-24

**Authors:** Bastian Schulze, Denice van Herwerden, Ian Allan, Lubertus Bijlsma, Nestor Etxebarria, Martin Hansen, Sylvain Merel, Branislav Vrana, Reza Aalizadeh, Bernard Bajema, Florian Dubocq, Gianluca Coppola, Aurélie Fildier, Pavla Fialová, Emil Frøkjær, Roman Grabic, Pablo Gago-Ferrero, Thorsten Gravert, Juliane Hollender, Nina Huynh, Griet Jacobs, Tim Jonkers, Sarit Kaserzon, Marja Lamoree, Julien Le Roux, Teresa Mairinger, Christelle Margoum, Giuseppe Mascolo, Emmanuelle Mebold, Frank Menger, Cécile Miège, Jeroen Meijer, Régis Moilleron, Sapia Murgolo, Massimo Peruzzo, Martijn Pijnappels, Malcolm Reid, Claudio Roscioli, Coralie Soulier, Sara Valsecchi, Nikolaos Thomaidis, Emmanuelle Vulliet, Robert Young, Saer Samanipour

**Affiliations:** 1 Queensland Alliance for Environmental Health Sciences (QAEHS), The University of Queensland, 202. Cornwall Street, QLD 4102 Woolloongabba, Australia; 2 Van ‘t Hoff Institute for Molecular Sciences (HIMS), University of Amsterdam, Science Park 904, 1098 XH Amsterdam, The Netherlands; 3 Norwegian Institute for Water Research (NIVA), Gaustadalléen 21, 0349 Oslo, Norway; 4 Environmental and Public Health Analytical Chemistry, Research Institute for Pesticides and Water, University Jaume I, Avda. Vincent Sos Baynat, s/n, 12071 Castelló de la Plana, Castellón Spain; 5 Plentzia Marine Station, Department of Analytical Chemistry, University of the Basque Country, Areatza Pasealekua, 48620 Plentzia, Basque Country Spain; 6 Aarhus University, Department of Environmental Science, Environmental Metabolomics Lab, Frederiksborgvej 399, 4000 Roskilde, Denmark; 7 INRAE, UR RiverLy, F-69625 Villeurbanne, France; 8 Masaryk University, Faculty of Science, RECETOX, Kamenice 753/5, 625 00 Brno, Czech Republic; 9 National and Kapodistrian University of Athens, Athens, Greece; 10 Vitens N.V., Oude Veerweg 1, Zwolle, 8001 BE The Netherlands; 11 Man-Technology-Environment Research Centre, School of Science and Technology, Örebro University, Fakultetsgatan 1, 701 82 Örebro, Sweden; 12 Eurolab Srl, Via Monsignore Rodolfi 22, IT-36022 Cassola, VI Italy; 13 Univ Lyon, CNRS, Université Claude Bernard Lyon 1, Institut des Sciences Analytiques, UMR 5280, 5 rue de la Doua, F-69100 Villeurbanne, France; 14 University of South Bohemia in České Budějovice, Faculty of Fisheries and Protection of Waters, South Bohemian Research Center of Aquaculture and Biodiversity of Hydrocenoses, Zátiší 728/II, CZ-389 25 Vodňany, Czech Republic; 15 Institut Català de Recerca de l’Aigua (ICRA) Catalan Institute for Water Research, Edifici H2O - Parc Científic i Tecnològic Universitat de Girona Carrer Emili Grahit, 101 E- 17003 Girona, Spain; 16 Eawag, Swiss Federal Institute of Aquatic Science and Technology, 8600 Duebendorf, Switzerland; 17 Univ Paris Est Creteil, Ecole des Ponts, LEESU, F-94010 Creteil, France; 18 Flemish Institute for Technological Research (VITO), Unit Separation and Conversion Technology, Boeretang 200, 2400 Mol, Belgium; 19 Department of Environment & Health, Faculty of Science, Amsterdam Institute of Molecular and Life Sciences, Vrije Universiteit Amsterdam, De Boelelaan 1085, 1081 Amsterdam, HV The Netherlands; 20 Department of Chemistry, University of Natural Resources and Life Sciences—BOKU Vienna, Muthgasse 18, 1190 Vienna, Austria; 21 Consiglio Nazionale delle Ricerche, Istituto di Ricerca Sulle Acque, Via De Blasio 5, 70132 Bari, Italy; 22 OSU-EFLUVE, Univ Paris Est Creteil, CNRS, F-94010 Creteil, France; 23 Department of Aquatic Sciences and Assessment, Swedish University of Agricultural Sciences (SLU), SE-75007, Uppsala, Sweden; 24 Ministry of Infrastructure and Water Management, Rijkswaterstaat, Zuiderwagenplein 2, 8224 Lelystad, AD Netherlands; 25 Instituto di Ricerca Sulle Acque, Consiglio Nazionale delle Ricerche, Via Mulino 19, IT- 20861 Brugherio, MB Italy; 26 BRGM, F-45060 Orleans, France; 27 Colorado State University, Soil and Crop Sciences Department, Plant Sciences C117, Fort Collins, CO 80523 United States

**Keywords:** Environmental monitoring, Mass spectrometry

## Abstract

Non-target analysis (NTA) employing high-resolution mass spectrometry is a commonly applied approach for the detection of novel chemicals of emerging concern in complex environmental samples. NTA typically results in large and information-rich datasets that require computer aided (ideally automated) strategies for their processing and interpretation. Such strategies do however raise the challenge of reproducibility between and within different processing workflows. An effective strategy to mitigate such problems is the implementation of inter-laboratory studies (ILS) with the aim to evaluate different workflows and agree on harmonized/standardized quality control procedures. Here we present the data generated during such an ILS. This study was organized through the Norman Network and included 21 participants from 11 countries. A set of samples based on the passive sampling of drinking water pre and post treatment was shipped to all the participating laboratories for analysis, using one pre-defined method and one locally (i.e. in-house) developed method. The data generated represents a valuable resource (i.e. benchmark) for future developments of algorithms and workflows for NTA experiments.

## Abstract (summary)

**Table:** 

Measurement(s)	chemical • drinking water
Technology Type(s)	high resolution mass spectrometry • non-target analysis • Interlaboratory
Factor Type(s)	method
Sample Characteristic - Environment	laboratory environment

Machine-accessible metadata file describing the reported data: 10.6084/m9.figshare.15028665

## Background & Summary

Non-target analysis (NTA) using high-resolution mass spectrometry (HRMS) is the most comprehensive approach for the screening and discovery of organic compounds/chemicals of emerging concern (CECs) in complex environmental samples^1–7^. This strategy is a bottom up approach with minimum a priori assumptions and/or knowledge regarding the samples and the CECs^5,8–10^. The recent surge in the number of newly identified CECs in different environmental compartments is a testimonial to the power of this technology^11–13^.

The main drawback of NTA is its complexity. Long and laborious processes increase the likelihood of false positive and false negative results, and the resulting data is difficult to interpret and can suffer from poor reproducibility^1,7,8,14–18^. Additionally, the wide variety of chemicals with different physico-chemical properties and variable concentration ranges make NTA an extremely challenging task^4,19^.

Several sophisticated algorithms and workflows have been developed in the past decade to tackle the complexity of NTA data^3,5,20–23^. Each of these approaches attempted to address one or more steps in the process, from noise removal, over peak picking to identification and finally communication of the confidence level of the identified CECs. While these algorithms have shown increasing success, there still remain some challenges with NTA and the underlying assumptions. Accordingly, recent studies have highlighted that further improvements are needed to be able to generate reproducible results^8,15,24,25^.

International collaborative efforts, such as raw data sharing (e.g. FAIR Principles^26,27^), novel CEC sharing (e.g. NormaNews^28^), community based spectral libraries (e.g. MassBank EU^29^ and MoNA), and former inter-laboratory studies (ILSs) have shown great potential in highlighting these shortfalls. Such work is crucial to steer future research and ensure success^9,12,13,21,28^. An example of such effort was the NTA collaborative trial organized by Norman Network^9,10^, which promoted a clear reporting strategy of the confidence levels in the identified CECs in complex samples within the environmental chemistry field^9,10^.

In this study, we present the data collected during an international collaborative ILS organized through the Norman Network. This study aimed at assessing the uncertainty in the identified compounds caused by different NTA workflows and spectral databases. We used a passive sampling (PS) strategy to sample river water at a drinking water intake, and post treatment for the production of drinking water. This will enable us to sample substances (i) present in river water at the drinking water intake, (ii) present in drinking water, as well identify those (iii) removed during treatment and (iv) generated during drinking water treatment.

PS enables to pre-concentrate chemicals from a complex matrix while leaving behind a significant proportion of unwanted matrix affecting the performance of the analysis, therefore allowing to sample chemicals often found at trace-levels in water and in the environment^30,31^. PS devices accumulate chemicals through diffusion over time when deployed in water (Table 1). When uptake kinetics are known and understood, it is possible to relate the amount of a chemical accumulated in a sampler to its time-averaged concentration in water for the period of deployment through a sampling rate, an equivalent volume of water extracted by the sampler per unit of time. This allows the collection of a more representative sample than when using spot sampling.

**Table 1:** Overview of samples provided to participants.

Vial number	Matrix type	Sampler Exposure time	Estimated sampled water volume	Code
Vial 1	PS extract – River water	2 days	4.8 L	S2 2
Vial 2	PS extract – River water	4 days	8.7 L	S2 4
Vial 3	PS extract – drinking water	2 days	4 L	S1 2
Vial 4	PS extract – drinking water	4 days	7.4 L	S1 4
Vial 5	Procedural blank (field blank)		—	B
Vial 6	RTI mixture standard		—	RTI

All participants in this ILS were given the same “ready for injection” samples consisting of the two surface water and two drinking water PS extracts. They were requested to analyze these PS extracts using one pre-defined method for liquid chromatography (LC) and mass spectrometry (MS), Table 2. Participants were also required to analyze the samples via their “in-house” methods. The participants were allowed the freedom to use any data processing strategy for the identification of CECs in those samples (details are provided in section Experimental Design).

**Table 2:** Gradient programme for the pre-defined method using a 150 mm × 2.1 mm column with 1.8 μm particles, with a flowrate of 0.4 ml/min.

Time	% A	% B
0	87	13
0.5	87	13
10.0	50	50
10.75	5	95
12.25	5	95
12.5	87	13
15.0	87	13

**Fig. 1:**
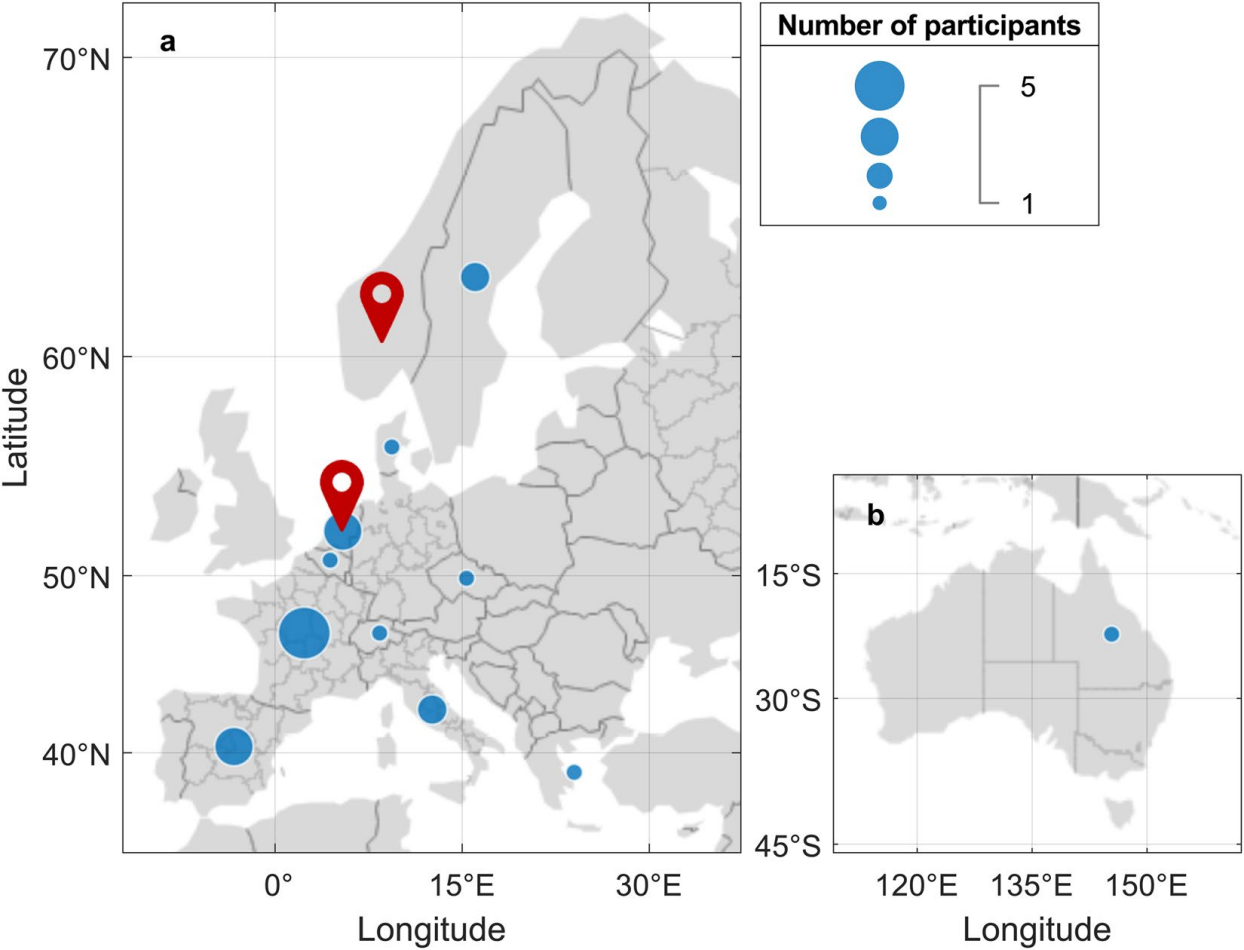
Map depicting the distribution of participating laboratories in the international ILS. The size of the bubble represents the number of participating labs in each country and the two organizing institutes of the ILS are represented by the red location markers. All laboratories providing data are located in Europe (**a**) except for one which is located in Australia (**b**).

This dataset includes the raw data from 21 different laboratories (Fig. 1), using different reversed-phase LC (RPLC) columns and instrumentations (Tables 3 and 4, respectively), chromatographic gradients, and MS acquisitions. This dataset provides the means to assess the impact of method transfer, chromatographic conditions, and data processing workflows on the results of NTA approaches. Additionally, this dataset may, potentially, be used as a benchmark for future development of algorithms for NTA workflows.

**Table 3:** Columns used for the pre-defined method and the in-house method, as indicated by the participant; the number of participants using the same column is indicated in brackets.

Manufacturer	Model pre-defined method	Model “In-house” method
Waters	Acquity (UPLC) BEH C18 (4)	Acquity (UPLC) BEH C18 (4)
	Acquity UPLC HSS C18 (2)	
	Acquity HSS-T3 C18	Acquity HSS-T3 C18
	Xselect HSS C18	Xselect HSS C18
	Xbridge C18	Xbridge C18
	Cortecs C18	Cortecs C18
	HSS T3 C18	HSS T3 C18
		Atlantis C18
Agilent	Zorbax Eclipse Plus C18	Zorbax Eclipse Plus C18
	Zorbax SB AQ	Zorbax SB AQ
	Zorbax Eclipse XDB C18	Zorbax Eclipse XDB C18
ThermoScientific	Hypersil BDS C18	
		Acclaim RSLC 120 C18 (2)
		Acclaim PepMap RSLC 100 C18
Phenomenex	Kinetex XB-C18	
	Luna C18 (3)	Luna C18
		Kinetex Biphenyl
		Kinetex EVO C18
Restek	Raptor C18	
ACE		UltraCore 2.5 SuperPhenylHexyl

**Table 4:** Manufacturers and models of MS instruments used by the participants.

Manufacturer	Model
Thermo Fisher Scientific	QExactive HF (2)
	QExactive Focus (2)
	QExactive (2)
	Orbitrap Lumos Fusion
	Orbitrap Velos
Waters	Xevo G2-S QTOF (3)
	Xevo G2 QTOF
	Xevo G2-XS QTOF
	Vion IMS Q-TOF
Bruker	Maxis Impact
	Maxis Plus
	Compact
Sciex	TripleTOF 5600+
	TripleTOF 6600
	X500R
Agilent	QTOF 6550

The number of participants using the same model of MS instruments is indicated in brackets.

## Methods

### Experimental design

The participants were given a set of samples and were asked to perform a complete NTA/extended suspect screening workflow (i.e. RPLC coupled with HRMS) using two different experimental approaches. A harmonized approach, hereafter referred to as pre-defined method (details are provided in the section “pre-defined method”) and an individually developed in-house method. Finally, the participants were requested to provide: the raw data (i.e. vendor format), the converted files (mzMXL format), the raw feature list associated with each sample, the top 50 identified features including the level of confidence in the identifications, based on the Schymanski scale^9^.

**Fig. 2:**
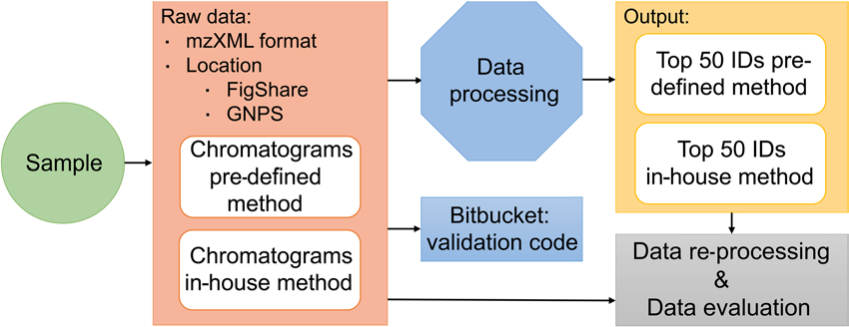
The experimental design of the ILS.

The experimental design (Fig. 2) allows the systematic assessment of the impact of the method transfer, chromatography approach, and data acquisition on the explored chemical space. Furthermore, it enables the evaluation of the impact of different data processing strategies on the identified features and the level of confidence associated to the identifications according to the tiered levels proposed by Schymanski *et al*.^9,10^.

### Sample set

A sample set including six vials was shipped to all 21 participants of this ILS. The vials consisted of four extracts from passive samplers (PS), one procedural blank, and a mixture of internal standards (IS) for the retention time modeling developed by the University of Athens (Table 1)^32^.

To generate the samples, integrative sampling at both the input and the output of a drinking water treatment plant (i.e. the source river water and drinking water, respectively) was carried out in June 2019 using Horizon Atlantic® HLB-L disks (47 mm diameter) with exposure times of 2 and 4 days. A detailed description of the sampling and sample preparation is given in supporting information (SI).

HLB disks were chosen as the receiving phase as the HLB sorbent has been shown to be suitable for the sampling of a variety of substances with different physicochemical properties, including compounds with low log*D*/*Kow* or ionized at river/drinking water pH^33,34^. However, limitations exist, particularly in the very low log*D* range.

In order to increase the sampling rate of the chemicals into the PS, the samplers were placed in a “dynamic” PS device (DPS), consisting of an electrically driven large volume water pumping device coupled to a PS exposure cell^35^. A total of 26 discs were exposed to treated drinking water and the same number to source water.

The exposed HLB disks, once in the lab, were kept in the freezer at −20 °C until extraction. The frozen disks were then freeze-dried for 48 hours to remove any water residues. The dried disks were spiked with six isotope-labeled ISs (i.e. Caffeine-13C3, Nicotine-D4, Cotinine-D3, Simazine-D10, Carbamazepine-D10, and Diuron-D6) prior to extraction. The spiking level was set at 50 ng/mL of each IS in the final extract, assuming a 100% recovery.

For the extraction, we used a previously tested wide-scope approach, which consisted of three consecutive extractions of the PS with 200 mL of acetone for 24 hr. The final extracts, then, were solvent exchange to methanol, following the US EPA method 3570^36^. The extracts of 13 different PS were pooled together to obtain homogeneous samples/extracts that were shipped to the participants. We also included a procedural blank, which consisted of the pooled extracts of seven PS brought to the field without being exposed to water.

In order to estimate the approximate volume of water sampled by the HLB disk PS, 0.5 mm thick silicone sheets made of AlteSil™ (Altec, UK) with the exposed surface area of 200 cm^2^ were co-deployed in the DPS next to the HLB disks. Prior to exposure, the silicone sheets were spiked with 14 performance reference compounds (PRCs)^35^. The loss of PRCs from silicone during exposure was applied to calculate the mass transfer coefficient (MTC) of PRCs through the water boundary layer (WBL). The estimated volumes of water extracted by HLB disks are based on the assumption of the same WBL-controlled MTC in silicone and HLB disks, by the approach shown by Vrana *et al*.^35^ and were 190 and 346 L for the river exposures for 2 and 4 days, 160 and 295 L for the drinking water exposures.

Considering the 40 times dilution used to prepare individual vials for distribution to participating laboratories, vials 1 to 4 contained equivalent volumes of water sampled by the HLB discs of 4.8, 8.7, 4.0 ad 7.4 L, respectively (Table 1). This can be applied to substances accumulated linearly over the exposure time by the HLB discs under boundary-layer controlled uptake. This is not necessarily applicable to all substances and samples.

### Pre-defined method

The pre-defined method consisted of two components, namely: 1) LC separation and 2) mass spectrometric data acquisition. The participants were given detailed instructions on how to set up the exact conditions on their instruments. Additionally, the participants were given access to a set of tools (e.g. method transfer and column chemistry assessment - https://find.waters.com/ColumnCoach/existingcolumn/column) to find the closest conditions to the instructions in the pre-defined method^37^.

1. Separation: the mobile phase consisted of 5 mM aqueous ammonium formate adjusted to pH 3 (A) and acetonitrile (ACN) containing 0.1% formic acid (B) used in a 15 min gradient elution (Table 2). The flow was given at 0.4 ml/min for a 150 mm × 2.1 mm × 1.8 μm C18 (Acquity UPLC BEH C18). The injection volume for this method was set to 5 µL. The pre-defined method was a wide scope generic method for analysis of pharmaceuticals and pesticides in environmental samples^16,38^.
2. Data acquisition: the participants were requested to run their samples in a data-independent acquisition mode. The mass range was set between 50 and 900 Da. For the quadrupole-time-of-flight (QTOF) instruments the sampling rate was limited to 2 Hz while for Orbitrap instruments the participants were advised to run their samples with maximum of 60,000 resolution. For the SWATH experiments, the number of windows was limited to 10. The method required acquisition in positive-polarity mode using an electrospray-ionization source. For the pseudo MS2 spectra, the participants were asked to perform their experiments either using a collision energy of 10–45 eV as a ramp or an average collision energy of 30–40 eV.

It should be noted that the observed diversity in the pre-defined method may or may not translate into the same level of diversity in the explored chemical space. In fact this can only be assessed by systematic re-processing of the data using open source/access workflows, which will be the subject of future studies by the consortium.

### Variations in the pre-defined method

Twenty out of the 21 participants, depending on the availability of instrumentations in their labs, implemented some changes in the pre-defined method, employing the provided tools by the organizers.

Four out of 21 participants opted for reconstitution of the extracts into ACN/water 50/50, ACN/water 14/86, methanol/water 10/90 or methanol, prior to the separation. One participant mixed the sample with mobile phase A (see above) at a 1:1 volume ratio.

As for the injection volumes, 16 participants followed the instructions in the pre-defined method (i.e. 5 µL) while three participants opted for 10 µL injection volume. One of the participants performed a large volume injection of 200 µL.

All participants used C18 columns for both the pre-defined and in-house methods. 14 out of 21 participants used the 0.4 mL/min flow rate suggested by the pre-defined method while six participants used different flow rates between 0.21 and 0.3 mL/min and one of 1 mL/min. Additionally, it should be noted that not all the participants employed the same column for both pre-defined and in-house methods (Table 3).

In terms of mass spectrometers, 8 Thermo Fisher Scientific, 6 Waters, 3 Bruker, 3 Sciex and 1 Agilent instruments were used for the data acquisition. All eight Thermo Fisher Scientific instruments were Orbitraps, while all other instruments relied on the time-of-flight (TOF) principle (Table 4). All the participants were asked to perform the analysis via data independent acquision (DIA). This acquisition mode of accurate-mass full-scan spectra under different collision induced dissociation conditions within a single injection is known under different names depending on the manufacturer: MSE in the case of Waters, broadband collision-induced dissociation (bbCID) in the case of Bruker, or all-ions MS/MS in the case of Agilent. Likewise for Q-Orbitrap instruments from Thermo, this type of acquisition is also possible, and known as All Ion Fragmentation (AIF) or variable Data-Independent Analysis (vDIA). An alternative DIA was Sequential Window Acquisition of All Theoretical Mass Spectra (*SWATH*-*MS*), which maily performed via AB Sciex instruments.

For the acquisition mode and collision energies, all participants followed the instructions provided. As for the mass range, 17 used the range defined in the pre-defined method whereas four participants used 75–950, 100–900, or 77–1000 Da.

### In-house method

The participants were also asked to analyze the extracts using an in-house method. Six out of 21 participants decided to reconstitute the sample in new solvent, five of which in methanol/water (4.8%, 10%, 20%, 50% and 100% methanol) and one in ACN/water 1/99. Injection volumes, in this case, ranged from 2 μL to 1000 μL, with 16 institutes using 5 μL, similar to the pre-defined method, and two using 10 μL.

Commonly applied flow rates ranged from 0.2 mL/min to 0.5 ml/min with the median at 0.375 mL/min while there were two cases with 1 mL/min and 0.0002 mL/min (nano LC). Two participants used flow gradients (0.2–0.48 ml/min and 0.3–0.4 ml/min).

As for column chemistries, we observed a greater diversity in the used columns when compared to the pre-defined method. More specialized columns were used for the in-house method. However, some participants used the same column for both methods (Table 3). The used mobile phases, varied mostly in the concentration of additives: water with ammonium acetate or formate (up to 10 mM) and formic acid (up to 0.1%) for mobile phase A and methanol or ACN, pure or with the same additives, for mobile phase B. All participants used gradients for elution, mostly running between 20 and 30 minutes.

The starting masses for the scan range ranged from 30 Da to 100 Da with a median of 60 Da, while the end of the scan range was between 800 and 1300 Da with the median at 925 Da. Collision energies were the same as the ones used for the pre-defined method except for slight changes (experimental details are provided in Record 3).

### Data conversion

The participants were asked to convert the datasets to mzXML^39^ format prior to submission using either the vendor provided tools or MSConvert implemented via ProteoWizard^40^. All the users employed the MSConvert 32− or 64-bit. Additionally, a minimum absolute intensity of 50 counts was applied to all mzXML files as the intensity threshold, in order to reduce the size of the converted data.

## Data Records

This ILS resulted in three different data records – i.e. mzXML files related to the pre-defined method, mzXML files related to the in-house method, and the experimental conditions in XLSX format.

### Record 1

includes the mzXML files for both the samples related to the river water and drinking water with exposure times of 2 and 4 days analyzed via the pre-defined method. This record includes 157 mzXML files^41,42^.

### Record 2

includes the mzXML files for both the samples related to the river water and drinking water with exposure times of 2 and 4 days analyzed via the own method. This record includes 134 mzXML files^42,43^.

### Record 3

consists of an excel file that includes the details of participants and the experimental conditions associated with each mzXML file, including the software packages used for the data acquisition and pre-processing^44^.

### Record GNPS

additionally, all the mzXML files from records 1 and 2 are also available for download or analysis via GNPS^42^.

## Technical Validation

We used the detection of IS, their mass accuracy, retention factor (i.e. run-time normalized retention time), and their intensity to assess the quality of the generated data by the participants. The extracted ion chromatograms (XIC) of the IS were generated using a mass window of ±0.01 Da. The peak of each IS was identified as the maximum signal in the selected XIC. Cases where we were not able to identify the IS signal as the most prominent signal, were considered as not detected. The measured mass (i.e. the median mass of three scans around apex), retention time, and intensity of apex were recorded for quality assurance of the data.

**Fig. 3:**
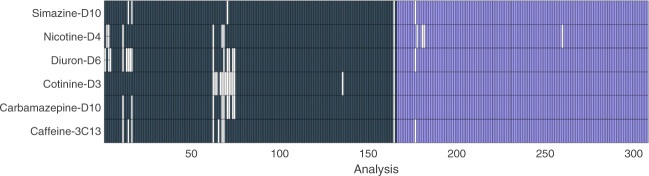
The detection overview for all six ISs for both pre-defined (dark gray) and in-house (light purple) methods. The white boxes are non-detected ISs.

**Fig. 4:**
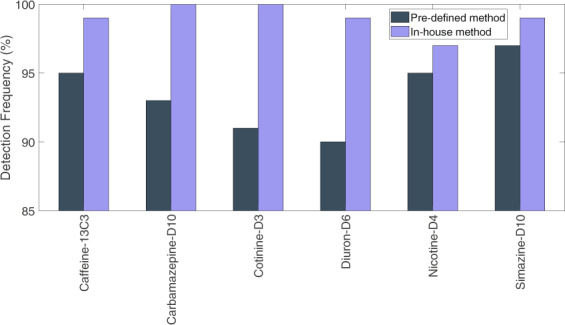
The detection frequency of each IS in pre-defined and in-house method.

**Fig. 5:**
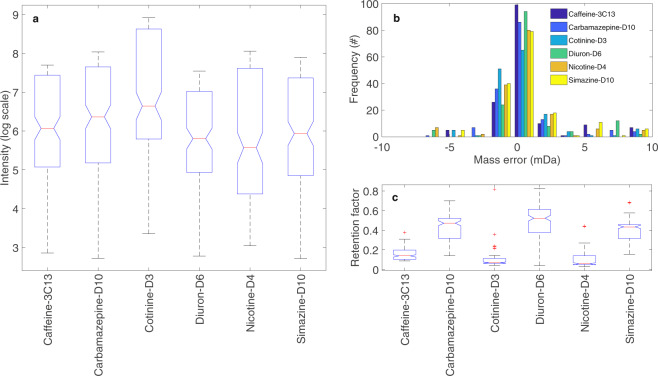
Variance observed in (**a**) the intensity in log scale, (**b**) the mass error (mDa), and (**c**) the retention factor of each IS recorded via pre-defined method. The retention factor was obtained by dividing the retention time for each detected IS by the total analysis time.

**Fig. 6:**
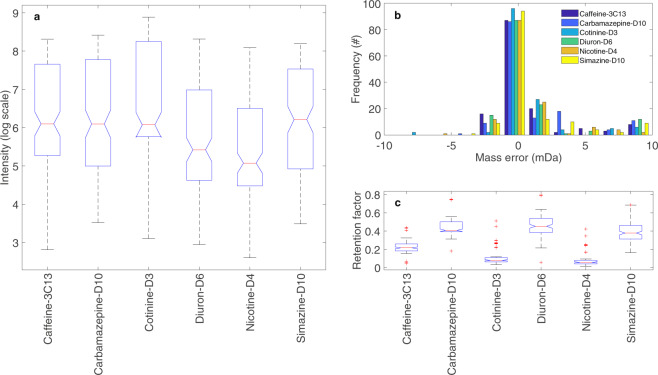
Variance observed in (**a**) the intensity in log scale, (**b**) the mass error (mDa), and (**c**) the retention factor of each IS recorded via in-house method. The retention factor was obtained by dividing the retention time for each detected IS by the total analysis time.

For 98% of datasets, the participants were able to detect all six IS, independently from the method used (i.e. pre-defined versus in-house). For two cases analyzed via the pre-defined method only three out of six ISs were detected while this was the case only for one chromatogram via own method, respectively. Higher detection frequencies were obtained for the in-house method than for the pre-defined method for all IS (Fig. 3). Diuron-D6 had the lowest detection frequency of 92% for pre-defined method whereas nicotine-D4 was the IS with the lowest detection frequency of 96% via the in-house method (Fig. 4). Diuron-D6 was the latest eluting IS with pre-defined method whereas nicotine-D4 was the IS with the smallest retention time for both methods (Figs. 5 and 6).

When looking at the mass error of IS, for both methods, a median of ±12 ppm was observed (Figs. 5 and 6). Overall, the mass errors were distributed between −7 mDa and +10 mDa, for both methods. We did not observe a statistically significant correlation between the retention time, m/z value and the measured mass error.

As for the intensities of the IS a median intensity of 10^6^ was measured across the datasets and the methods. The variance in the intensities of around five orders of magnitude appeared to be independent from the method used for the analysis.

## Usage Notes

Often, when developing new tools for NTA, they are restricted to a limited number of datasets usually generated in one laboratory. These datasets rarely represent the actual diversity in potential future datasets that should be processed using these tools. In this data descriptor, we present a dataset which covers a wide range of instruments and instrumental/analysis conditions. Additionally, the dataset in this study provides three different levels of chemical complexity (i.e. the number of potential chemical constituents) in blank, drinking water, and river water, respectively. Such a dataset can be used for the assessment of the impact of method transfer on the explored chemical space of the samples as well as the quality of the generated spectra. The records made available with this data descriptor constitute a valuable resource for the future development of NTA algorithms and workflows, for example by providing a collection of successfully identified compounds and retention time indices obtained over a wide range of instruments and analytical conditions. Altogether, these can be used by individual laboratories for evaluation of their own practices.

When using this data, the records 1 and 2 have the following name structure: InstituteID_(nr)_pd.mzXML for the pre-defined method and InstituteID_(nr)_own.mzXML for the in-house method. These files can be opened with almost any open source software for MS data.

## Supplementary information

Suplementary Information

## Data Availability

The script for the extraction of the IS signal and the plots in this data descriptor is available in this repository: https://bitbucket.org/Denice_van_Herwerden/ils-validation/src/main/.
