# Supplementary material for: Inter-laboratory mass spectrometry dataset based on passive sampling of drinking water for non-target analysis
Source: Sci Data. 2021 Aug 24;8:223. doi: 10.1038/s41597-021-01002-w (PMC8384892; doi:10.1038/s41597-021-01002-w)
Supplement: Supplementary file 1 — Suplementary Information [file 41597_2021_1002_MOESM1_ESM.pdf]

Supporting Information for: Inter-laboratory high resolution mass spectrometry dataset based on passive sampling of drinking water for non-target analysis

Authors

Bastian Schulze[1], Denice van Herwerden[2], Ian Allan[3], Lubertus Bijlsma[4], Nestor Etxebarria[5], Martin Hansen[6], Sylvain Merel[7], Branislav Vrana[8], Reza Aalizadeh[9], Bernard Bajema[10], Florian Dubocq[11], Gianluca Coppola[12], Aurélie Fildier[13], Pavla Fialová [8], Emil Frøkjær[6], Roman Grabic[14], Pablo Gago-Ferrero[15], Thorsten Gravert[6], Juliane Hollender[16], Nina Huynh[17], Griet Jacobs[18], Tim Jonkers[19], Sarit Kaserzon[1], Marja Lamoree[19], Julien Le Roux[17], Teresa Mairinger[16,20], Christelle Margoum[7], Giuseppe Mascolo[21], Emmanuelle Mebold[22], Frank Menger[23], Cécile Miège[7], Jeroen Meijer[19], Régis Moilleron[17], Sapia Murgolo[21], Massimo Peruzzo[13], Martijn Pijnappels[24], Malcolm Reid[3], Claudio Roscioli[25], Coralie Soulier[26], Sara Valsecchi[25], Nikolaos Thomaidis[9], Emmanuelle Vulliet[11], Robert Young[6,27], Saer Samanipour[2,3]

Affiliations

1. Queensland Alliance for Environmental Health Sciences (QAEHS), The University of Queensland, 202. Cornwall Street, QLD 4102, Woolloongabba, Australia.
2. Van ‘t Hoff Institute for Molecular Sciences (HIMS), University of Amsterdam, Science Park 904, 1098 XH, Amsterdam, The Netherlands.
3. Norwegian Institute for Water Research (NIVA), Gaustadalléen 21, 0349, Oslo, Norway.
4. Environmental and Public Health Analytical Chemistry, Research Institute for Pesticides and Water, University Jaume I, Avda. Vincent Sos Baynat, s/n, 12071 Castelló de la Plana, Castellón, Spain.
5. Plentzia Marine Station, Department of Analytical Chemistry, University of the Basque Country, Areatza Pasealekua, 48620 Plentzia, Basque Country, Spain.
6. Aarhus University, Department of Environmental Science, Environmental Metabolomics Lab, Frederiksborgvej 399, 4000 Roskilde, Denmark.
7. INRAE, UR RiverLy, F-69625 Villeurbanne, France.
8. Masaryk University, Faculty of Science, RECETOX, Kamenice 753/5, 625 00 Brno, Czech Republic.
9. National and Kapodistrian University of Athens, Athens, Greece.
10. Vitens N.V., Oude Veerweg 1, Zwolle, 8001 BE, the Netherlands.
11. Man-Technology-Environment Research Centre, School of Science and Technology, Örebro University, Fakultetsgatan 1, 701 82 Örebro, Sweden.
12. Eurolab Srl, Via Monsignore Rodolfi 22, IT-36022 Cassola, VI, Italy.
13. Univ Lyon, CNRS, Université Claude Bernard Lyon 1, Institut des Sciences Analytiques, UMR 5280, 5 rue de la Doua, F-69100 VILLEURBANNE, France.
14. University of South Bohemia in České Budějovice, Faculty of Fisheries and Protection of Waters, South Bohemian Research Center of Aquaculture and Biodiversity of Hydrocenoses, Zátiší 728/II, CZ-389 25 Vodňany, Czech Republic

15. Institut Català de Recerca de l'Aigua (ICRA) Catalan Institute for Water Research, Edifici H2O - Parc Científic i Tecnològic Universitat de Girona Carrer Emili Grahit, 101 E- 17003 Girona (Spain).
16. Eawag, Swiss Federal Institute of Aquatic Science and Technology, 8600 Duebendorf, Switzerland.
17. Univ Paris Est Creteil, Ecole des Ponts, LEESU, F-94010 Creteil, France.
18. Flemish Institute for Technological Research (VITO), Unit Separation and Conversion Technology, Boeretang 200, 2400 Mol, Belgium
19. Department of Environment & Health, Faculty of Science, Amsterdam Institute of Molecular and Life Sciences, Vrije Universiteit Amsterdam, De Boelelaan 1085, 1081 HV Amsterdam, The Netherlands.
20. Department of Chemistry, University of Natural Resources and Life Sciences—BOKU Vienna, Muthgasse 18, 1190 Vienna, Austria.
21. Consiglio Nazionale delle Ricerche, Istituto di Ricerca Sulle Acque, Via De Blasio 5, 70132, Bari, Italy.
22. OSU-EFLUVE, Univ Paris Est Creteil, CNRS, F-94010 Creteil, France.
23. Department of Aquatic Sciences and Assessment, Swedish University of Agricultural Sciences (SLU), SE-75007 Uppsala, Sweden.
24. Ministry of Infrastructure and Water Management, Rijkswaterstaat, Zuiderwagenplein 2, 8224 AD Lelystad, Netherlands.
25. Instituto di Ricerca Sulle Acque, Consiglio Nazionale delle Ricerche, Via Mulino 19, IT-20861 Brugherio, MB, Italy.
26. BRGM, F-45060 Orleans, France.
27. Colorado State University, Soil and Crop Sciences Department, Plant Sciences C117, Fort Collins, CO 80523, United States.

corresponding author(s): Saer Samanipour (s.samanipour@uva.nl)

Passive sampling method description

HLB disk passive samplers

To generate the samples necessary for this ILS, integrative sampling at both the input and the output of a drinking water treatment plant (i.e. the source river water and drinking water, respectively) was carried out using Horizon Atlantic® HLB-L disks (Biotage AB, Sweden) with 47 mm diameter (further denoted as HLB disks), applied as diffusive passive samplers. Before exposure, HLB disks were cleaned in acetone, isopropanol,methanol and milliQ water, in which they were stored at 4 °C. One tested sample comprised pooled extract of 13 exposed HLB disks or 7 HLB disks applied as a field blank.

Silicone sheets for estimation of sampled water volume

In order to estimate the approximate volume of water sampled, silicone elastomer sheets SSP250 (Speciality Silicone Products, Inc.; 250µm thick) were cut into 9.5×5.5 cm, Soxhlet extracted in ethylacetate for 72 h and spiked according to the procedure described in

(Smedes and Booij, 2012) with 14 performance reference compounds (PRC: IUPAC PCB congeners 1, 2, 3, 10, 14, 21, 30, 50, 55, 78, 104, 145 and 204) at 5–75 µg mL^{-1} in ethyl acetate was kindly provided by Deltares, Utrecht, the Netherlands. One sampler comprised 4 sheets with an exposed surface area of 200 cm^2 surface are (one sided) and a mass of 6 g.

Dynamic sampling device

In order to increase the sampling rate of the chemicals into the PS, the PS were exposed in a "dynamic" passive sampling device (DPS). The DPS device consists of a rectangular stainless-steel plate chamber with an open grid on both sides. The different samplers were placed on the grid and covered by lids. One end of the chamber was connected to a submersible pump (approximately 9 m^3 h^{-1}) that forced water at high flow velocity (1–2 m s^{-1}) through the chamber while being immersed in the water. Temperature was monitored by a submersible logger (Hobo Pendant, Onset, Germany) attached to the DPS device. Details of the DPS construction are provided in (Vrana et al., 2018). Note that the stated sampler exposure surface area was nominal, while in practice 80% had contact with water and ~20% was covered by the steel grid holding them in place inside the DPS.

Deployment and retrieval

Water temperature was 16.5 and 15.9°C in drinking and river water, respectively. pH of the sampled water was 7.85 and 7.61 in drinking water and river water, respectively.
Samplers were always mounted in the DPS device just before exposure and retrieved immediately afterwards. Recovered samplers were placed back into their storage containers, stored at 4 °C, transported to the laboratory immediately, and stored at −20 °C until further processing. To estimate any contaminant uptake not associated with water exposure, field blank samplers were exposed to air in a stainless-steel tray during sampler's mounting and retrieval.

Analysis of PRCs in silicone samplers

PRC amounts in silicone samplers were analysed using thermal desorption coupled online with gas chromatography mass spectrometry (GC/MS). Two 5-mg pieces (app. 5 mm in diameter) were cut from the middle of each silicone sheet, including both exposed and unexposed reference sheets. Silicone piece from each sampler were then directly inserted into thermal desorption liners for analysis of PRCs by thermal desorption followed by GC/MS as described in (Vrana et al., 2016). Following the thermal desorption, exact mass of a piece was weighed and used for determination of initial concentration of PRCs in reference sheets ($C_{0,PRC}$) and PRC concentration retained in following exposure in DPS (C_{PRC}).

Estimation of water boundary layer-controlled sampling rates of silicone samplers

The sampling rate of compounds for the silicone samplers ($R_{S,SR}$) was calculated from the fraction *f* of PRC concentrations retained in the sampler following exposure ($C_{PRC}/C_{0,PRC}$). $R_{S,SR}$ was modelled as a function of the molar mass (M) by the water boundary layer (WBL)-controlled uptake model from (Rusina et al., 2010).

$$R_{S,SR} = FM^{-0.47} \qquad (1)$$

with an exposure-specific parameter (*F*). The parameter *F* was estimated from the percentage of PRC dissipation from samplers during exposure using a nonlinear least squares method by (Booij and Smedes, 2010), considering the fractions of individual PRCs that are retained in the sampler after exposure as a continuous function of their sampler-water partition coefficient ($K_{SR,W}$). The models applied for silicone samplers are described in detail in (Vrana et al., 2018).

Estimation of sampled water volumes for HLB samplers

The estimated volume of water extracted by HLB disks (Table 1) is based on the assumption of fully time-integrative uptake and similar WBL-controlled mass transfer coefficient of small organic molecules in vicinity of silicone and HLB disks, by the approach demonstrated in (Vrana et al., 2018).
The sampling rates of ED samplers Rs,HLB were estimated from sampling rates derived for SR samplers (Rs,SR), using d the surface areas of both samplers A_{HLB}, A_{SR}:

$$R_{S,HLB} = \frac{A_{HLB}}{A_{SR}} R_{S,SR} \qquad (2)$$

The WBL-controlled sampling rate estimate $R_{S,HLB}$ obtained here should be from theory (Booij et al., 2007) a function of the compound's diffusion coefficient in water and can be estimated for any compound from its molar mass *M* using Eq. (1). The $R_{S,HLB}$ value of amodel compound with M = 300 g mol^{-1}, calculated using Eq. (1), was used for estimation of WBL-controlled sampling rates $R_{S,HLB}$ using Eq. (2). Further assuming a time integrative sampling, approximate sampled water volumes in HLB disks (Table 1 in the main manuscript), as:

$$V_{S,HLB} = R_{S,HLB} t \qquad (3)$$

Table S1. Passive sampler performance parameters.

**Vial number**	**Matrix type**	**Sampler Exposure time**	**Code**	**Silicone sampling rate $R_{S,SR}$ (L/d)**	**Estimated sampled water volume[a]**
Vial 1	River water	2 days	S2 2	82	190 L
Vial 2	River water	4 days	S2 4	74	346 L
Vial 3	Drinking water	2 days	S1 2	71	160 L
Vial 4	Drinking water	4 days	S1 4	65	295 L

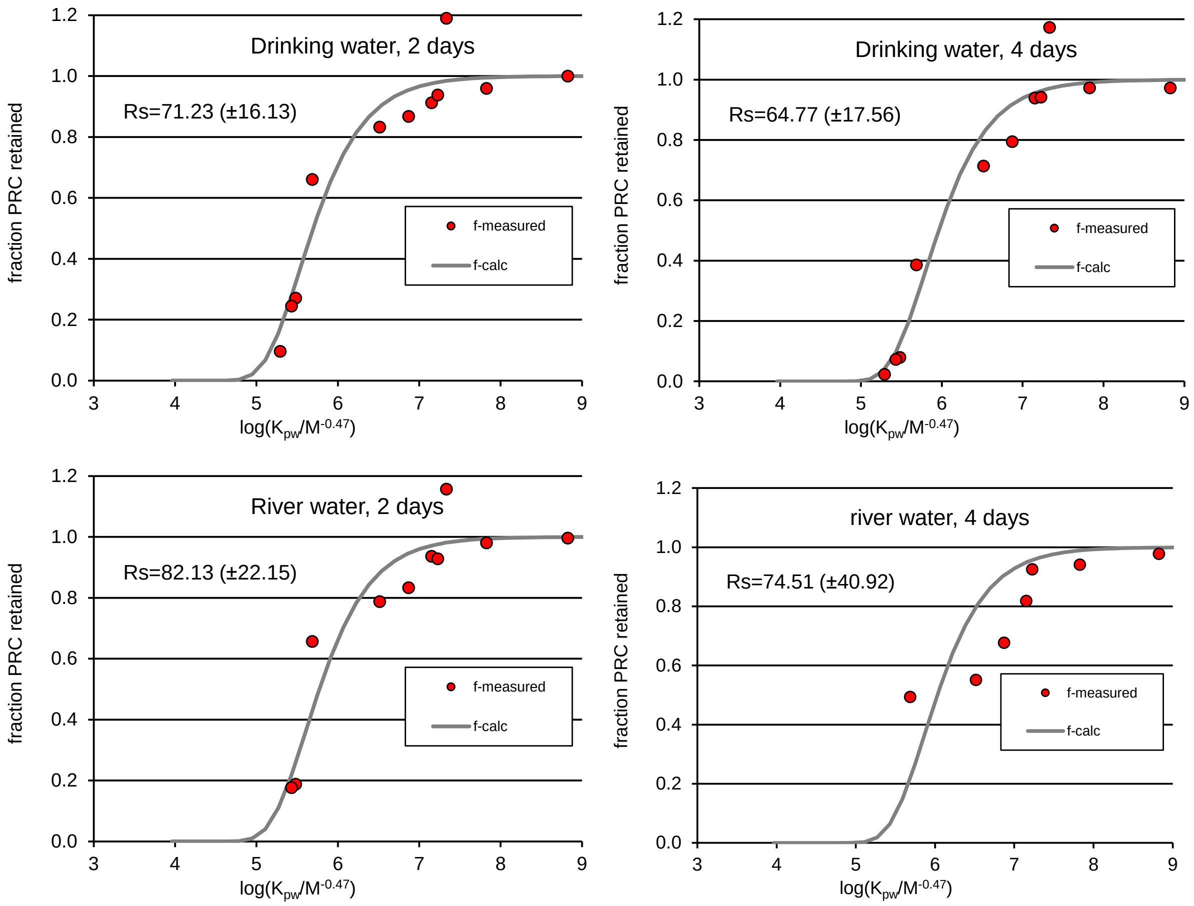

Fig S1. Water boundary layer-controlled R_S estimation from PRC dissipation from silicone samplers co-deployed in DPS device with HLB disks. The fraction of PRCs retained in the silicone sheets after exposure (Y-axis of graphs) were fitted by a continuous function of their K_{PW} and molar mass M. The drawn lines represent the best nonlinear least- squares fit of the data. The number in the graph shows the $R_{S,SR}$ (L d^{-1}) for a model compound with a molar mass of 300 g mol^{-1}.

References

Booij, K., Smedes, F., 2010. An improved method for estimating in situ sampling rates of nonpolar passive samplers. Environ. Sci. Technol. 44, 6789–94. https://doi.org/10.1021/es101321v

Booij, K., Vrana, B., Huckins, J.N., 2007. Theory, modelling and calibration of passive samplers used in water monitoring, in: Greenwood, R., Mills, G., Vrana, B. (Eds.), Comprehensive Analytical Chemistry 48. Passive Sampling Techniques in Environmental Monitoring. Elsevier, Amsterdam, pp. 141–169. https://doi.org/10.1016/S0166-526X(06)48007-7

Rusina, T.P., Smedes, F., Koblizkova, M., Klanova, J., 2010. Calibration of Silicone Rubber Passive Samplers: Experimental and Modeled Relations between

Sampling Rate and Compound Properties. Environ. Sci. Technol. 44, 362–367. https://doi.org/10.1021/es900938r

Smedes, F., Booij, K., 2012. Guidelines for passive sampling of hydrophobic contaminants in water using silicone rubber samplers. Ices Tech. Mar. Environ. Sci. 52, 20.

Vrana, B., Komancová, L., Sobotka, J., 2016. Calibration of a passive sampler based on stir bar sorptive extraction for the monitoring of hydrophobic organic pollutants in water. Talanta 152, 90–97. https://doi.org/10.1016/j.talanta.2016.01.040

Vrana, B., Smedes, F., Allan, I., Rusina, T., Okonski, K., Hilscherová, K., Novák, J., Tarábek, P., Slobodník, J., 2018. Mobile dynamic passive sampling of trace organic compounds: Evaluation of sampler performance in the Danube River. Sci. Total Environ. 636, 1597–1607. https://doi.org/10.1016/j.scitotenv.2018.03.242
